# Cysteamine broadly improves the anti-plasmodial activity of artemisinins against murine blood stage and cerebral malaria

**DOI:** 10.1186/s12936-016-1317-3

**Published:** 2016-05-06

**Authors:** Neda Moradin, Sabrina Torre, Susan Gauthier, Mifong Tam, Jalal Hawari, Kirsten Vandercruyssen, Bart De Spiegeleer, Anny Fortin, Mary M. Stevenson, Philippe Gros

**Affiliations:** Department of Biochemistry, McGill University, 3649 Promenade Sir William Osler, room 366, Montreal, QC H3G 0B1 Canada; Department of Human Genetics, McGill University, Montreal, Canada; Research Institute of the McGill University Health Centre, Montreal, Canada; Department of Civil, Geological and Mining Engineering, Ecole Polytechnique, Université de Montreal, Montreal, Canada; Faculty of Pharmaceutical Sciences, Ghent University, Ghent, Belgium

**Keywords:** *Plasmodium*, Blood-stage malaria, Cerebral malaria, Cysteamine, Artemisinins, Drug resistance

## Abstract

**Background:**

The potential emergence and spread of resistance to artemisinins in the *Plasmodium falciparum* malaria parasite constitutes a major global health threat. Hence, improving the efficacy of artemisinins and of artemisinin-based combination therapy (ACT) represents a major short-term goal in the global fight against malaria. Mice defective in the enzyme pantetheinase (*Vnn3*) show increased susceptibility to blood-stage malaria (increased parasitaemia, reduced survival), and supplementation of *Vnn3* mutants with the reaction product of pantetheinase, cysteamine, corrects in part the malaria-susceptibility phenotype of the mutants. Cysteamine (Cys) is a small, naturally occurring amino-thiol that has very low toxicity in vivo and is approved for clinical use in the life-long treatment of the kidney disorder nephropathic cystinosis.

**Methods:**

The ability of Cys to improve the anti-plasmodial activity of different clinically used artemisinins was tested. The effect of different CYS/ART combinations on malarial phenotypes (parasite blood-stage replication, overall and survival from lethal infection) was assessed in a series of in vivo experiments using *Plasmodium* strains that induce either blood-stage (*Plasmodium chabaudi* AS) or cerebral disease (*Plasmodium berghei* ANKA). This was also evaluated in an ex vivo experimental protocol that directly assesses the effect of such drug combinations on the viability of *Plasmodium* parasites, as measured by the ability of tested parasites to induce a productive infection in vivo in otherwise naïve animals.

**Results:**

Cys is found to potentiate the anti-plasmodial activity of artesunate, artemether, and arteether, towards the blood-stage malaria parasite *P. chabaudi* AS. Ex vivo experiments, indicate that potentiation of the anti-plasmodial activity of artemisinins by Cys is direct and does not require the presence of host factors. In addition, potentiation occurs at sub-optimal concentrations of artemisinins and Cys that on their own have little or no effect on parasite growth. Cys also dramatically enhances the efficacy and protective effect of artemisinins against cerebral malaria induced by infection with the *P. berghei* ANKA parasite.

**Conclusion:**

These findings indicate that inclusion of Cys in current formulations of ACT, or its use as adjunct therapy could improve the anti-plasmodial activity of artemisinin, decrease mortality in cerebral malaria patients, and prevent or delay the development and spread of artemisinin resistance.

**Electronic supplementary material:**

The online version of this article (doi:10.1186/s12936-016-1317-3) contains supplementary material, which is available to authorized users.

## Background

Malaria is a severe threat to global health with an estimated 300–500 million cases annually and >1 million deaths. In endemic areas of Africa, malaria accounts for 25 % of paediatric deaths [1, 2]. *Plasmodium falciparum* is by far the deadliest of the four human malarial species (*P. falciparum*, *Plasmodium malariae*, *Plasmodium ovale*, and *Plasmodium vivax*). The clinical symptoms of malaria occur at the blood stage when parasites rapidly replicate and lyse red blood cells (RBCs) leading to anemia and high fever. In addition, parasitized RBCs can become trapped and cause lesions in brain capillaries resulting in cerebral malaria (CM), the most lethal form of the disease [3].

Treatment of clinical malaria relies on a handful of drugs, the most potent being artemisinin derivatives (called the artemisinins or ADs), a group of structurally related molecules that includes artesunate (ART), artemether (ARTM) and arteether (ARTE) [4–6]. To avoid appearance and spread of artemisinin resistance in *P. falciparum*, the World Health Organization (WHO) has strongly discouraged use of artemisinins as a single agent, and artemisinin monotherapy is only recommended for lethal cerebral malaria [3]. Rather, and for all other forms of clinical disease, artemisinins are administered in combination (artemisinin combination therapy; ACT) with other, long acting, anti-malarial drugs that include mefloquine, lumefantrine, amodiaquine, piperaquine and sulfadoxine/pyrimethamine [4, 5]. Because no other drugs as potent as artemisinins are available, the potential emergence of artemisinin resistance at Thai/Cambodia border and its spread to other areas has caused significant concerns [7–11], including the failure of front line ACT due to secondary partner drug resistance. Although the mechanisms of artemisinin resistance in *Plasmodium* are being characterized [12–16], novel adapted treatment options based on this knowledge are years away. Hence, improving the efficacy of artemisinins and of ACT represents a major short-term goal in treating and preventing the spread of artemisinin resistance in the malaria parasite. Likewise, increasing effectiveness of adjunct treatment to artemisinin monotherapy may improve the outcome of cerebral malaria, the most severe and most difficult to treat complication of malaria.

Studies in human populations from areas of endemic disease have long established the critical role of genetic factors in susceptibility and protection against malaria [17, 18]. Examples include the protective effect of heterozygosity for loss of function variants at erythrocyte-specific proteins such as haemoglobin (sickle cell anaemia, thalassaemias), glucose 6-phosphate dehydrogenase, anion exchanger 1 (SLC4A1), Duffy antigen (DARC), ABO blood group variants and several others [17, 19]. Likewise, studies in mouse models of blood stage malaria (*Plasmodium chabaudi* AS), and of cerebral malaria (*Plasmodium berghei* ANKA) have also established a strong genetic control of resistance and susceptibility to malaria, and the molecular basis has been characterized in several instances [20], providing potentially useful entry points for discovery of novel anti-malarial drugs or other treatment modalities [21, 22].

In a mouse model of infection with *P. chabaudi*, it was reported that a loss of function in the enzyme pantetheinase (*Vnn1/Vnn3*) in mouse strain AcB61 causes susceptibility to blood-stage malaria [23]. The *Vnn1/Vnn3* pantetheinases are enzymes that hydrolyze pantetheine to pantothenic acid (vitamin B5) and Cysteamine (Cys). Furthermore, Cys displays modest but significant anti-malaria activity, and Cys treatment can significantly improve the response of mice to blood stage infection with *P. chabaudi* (reduced parasitaemia, increased survival), when given either as a prophylactic (naïve animals) or as a therapeutic (infected animals) regimen [24]. Ex vivo, Cys inhibits the degradation of hemoglobin by *Plasmodium* parasites in erythrocytes [24].

Cys is a small, naturally occurring amino thiol that has very low toxicity in vivo. Importantly, different Cys formulations are approved for life-long treatment of nephropathic cystinosis (NC), a kidney disorder caused by mutations in the lysosomal cystine carrier cystinosin [25]. It was reported that Cys dosing regimens that display pharmacokinetic profiles similar to those measured in humans taking oral Cys for the treatment of NC, reduce replication of the malaria parasite in vivo, and reduce lethality from infection [26]. In the present study, the potential of Cys to increase the anti-plasmodial activity of different artemisinins towards the murine *Plasmodium* parasites *P. chabaudi* AS (blood-stage), and *P. berghei* ANKA (cerebral malaria) was investigated. Cys is shown to potentiate the activity of several artemisinins currently in clinical use in ACT, including ART, ARTE, and ARTM, against the blood stage of the infection by *P. chabaudi* in mice. Cys also causes dramatic enhancement of artemisinin efficacy and protective effect against cerebral malaria induced in the mouse by infection with the ANKA strain of *P. berghei*.

## Methods

### Mice and parasites

Eight to twelve weeks old female A/J mice were purchased from the Jackson Laboratories (Bar Harbor, ME, USA) and were housed at McGill University Animal Care Center according to the guidelines of the Canadian Council on Animal Care. Mice experimentation protocol was approved by the McGill Facility Animal Care Committee (P. Gros, Principal Investigator; protocol number: 5287), and include procedures to minimize distress and improve welfare. An isolate of *P. chabaudi* AS was provided by Dr. Mary M. Stevenson (McGill University Health Center Research Institute, Montreal), and was maintained by weekly passage in A/J mice for a maximum of four consecutive passages, as previously described [27]. *Plasmodium berghei* ANKA parasites were initially obtained from the Malaria Reference and Research Reagent Resource Center (MR4); *P. berghei* was passaged in C57BL/6J (B6) mice for a maximum of three consecutive passages. In passage mice, blood parasitaemia was monitored daily, and the percentage of parasitized red blood cells (pRBCs) was determined on thin blood smears stained with Dif-Quik (Dade Behring, Newark, DE, USA), as described [28]. Infected blood was diluted in pyrogen-free saline for infection of experimental animals and also for preparation of cryo-preserved stocks for long-term storage.

### Drugs and drug treatments

Cysteamine hydrochloride (Cys) and N-acetyl cysteine (NAC) (Sigma, Burlington ON, Canada) were prepared fresh in PBS. Artesunate and artemether were generously provided by Dafra Pharma International (Turnhout, Belgium). Artesunate was prepared fresh as a 1 mg/mL stock in 5 % sodium bicarbonate solution which was diluted in PBS immediately before use. Artemether (Artesiane™) prepared from fractionated coconut oil stock (20 mg/mL, injectable) was diluted in sesame oil to desired concentration. Beta-Arteether (Artecef™, Artemotil; 50 mg/mL injectable in sesame oil) was diluted in sesame oil to desired concentration. Beta-Arteether was generously provided by Artecef BV (Zeewolde, The Netherlands). Drugs were administered via intraperitoneal (i.p) injection and according to a four-days treatment schedule. Mice were weighed prior to treatment to determine appropriate doses and injection volumes ranged from 100–400 μL per mouse. In the case of animals treated with two drugs, Cys was administered first, followed by artemisinin derivatives 15 min later on alternate sides of the mouse abdomen. Untreated control animals were injected with PBS alone.

### Infections

For in vivo infections, mice were infected i.v. (0.2 mL) with 10^7^*P. chabaudi* pRBCs into the tail vein, and monitored under BSL2 containment conditions. One hour post-infection, mice received either PBS or different drugs or drug combinations (i.p), and this treatment was repeated at 24, 48, and 72 h post-infection. At day 4 and 5 post-infection, blood was sampled, and blood parasitaemia was determined (between 4–10 fields of 100 erythrocytes were counted per mouse, and results are expressed as percentage of parasitized erythrocytes). For experiments involving ex vivo drug exposure followed by in vivo infections, parasitized erythrocytes were exposed to certain compounds for 1 h at 37 °C. Treated pRBCs were then rinsed free of drug, and resuspended in drug-free PBS (at 1 × 10^6^ pRBC/mL), and were then used to infect naïve mice (1 × 10^5^ pRBC). The ability of the treated parasites to induce a productive infection was monitored by measuring blood parasitaemia daily, and by monitoring appearance and progression of lethal and irreversible symptoms of cerebral malaria, as we described [29].

### Pharmacokinetic studies of artemisinin in vivo

Mice were injected with Cys (140 mg/kg, 0.2 mL, i.p.) in the lower left abdomen, and exactly 15 min later, ART (0.5 mg/kg, 0.2 mL, i.p.) was injected in the lower right abdomen. At 5, 10, and 20 min, mice were euthanized and bled by cardiac puncture and blood was collected in EDTA-containing tubes to isolate plasma. Plasma samples showing hemolysis were not used for ART determination as haemolysis interfered with ART measurement. Control plasma from untreated mice was also included as a placebo for spiked calibration references covering a range between 5 and 2000 ng/mL for ART as well as DHA, and QC samples (10 and 100 ng/mL). The method consisted of a sample preparation step, followed by UPLC with MS (MRM-mode) detection. Briefly, a protecting solution consisting of potassium oxalate and sodium fluoride was added to ice-cold mouse plasma, followed by the addition of the ART-d3 and DHA-d3 internal standard solutions. After mixing, the diluted plasma samples were loaded on preconditioned µ-HLB-SPE wells, which are washed with water followed by 5 % acetonitrile in water. Elution of the compounds was done with a methanol–acetonitrile mixture, and diluted with water before injection in the UPLC system, consisting of an Acquity BEH shield RP18 column with a mobile phase consisting of water-acetonitrile-formic acid (50/50/0.1) in isocratic mode. The MRM transitions used for quantification were m/z 283→265, resp 286→268, for ART and its internal standard, and m/z 323→240, resp 326→243, for DHA and its internal standard. The method was validated and found to comply for linearity, selectivity, recovery, accuracy and precision.

### Evans blue dye extravasation

*Plasmodium berghei* parasites were subjected to ex vivo drug exposure followed by intravenous injection in B6 mice. At day 6 post-infection, mice were injected intravenously with 0.2 mL 1 % Evans blue dye prepared in sterile PBS. One hour later, mice were exsanguinated and perfused with PBS-containing 2 mM EDTA. Brains were excised, imaged, and incubated with 1 mL of dimethyl formamide for 48 h to extract Evan’s blue dye from the tissues. Optical density was measured at 610 nm, and measurements were converted into µg of dye extravasated per gram of tissue.

### Leukocyte infiltration in the brain

Six days post-infection, mice were exsanguinated and perfused as above; brains were harvested and homogenized in RPMI media-containing 0.5 mg/mL collagenase (Gibco LifeTechnologies), 0.01 mg/mL DNAse I (Roche) and 2 mM EDTA. Infiltrating cells were separated on a 33.3 % Percoll solution. Cells were stained for flow cytometry analyses with the following; Zombie Aqua Dye-V500 (BioLegend), anti-CD45-APC-eFluor780 (clone 30-F11, eBioscience), anti-CD4-PerCP Cyanine5.5 (clone RM4-5, eBioscience), anti-CD8alpha-PE (clone 53-6.7, eBioscience), anti-TCRbeta-FITC (clone H57-597, eBioscience), anti-CD19-BV421 (clone 6D5, BioLegend), F4/80-eFluor450 (clone BM8, eBioscience), anti-CD11b-APC (clone M1/70, eBioscience), anti-CD11c-PerCP Cyanine5.5 (clone N418, eBioscience), anti-Ly6C-PE (clone HK1.4, eBioscience), and anti-Ly6G-FITC (clone 1A8, BioLegend). Leukocytes were gated as CD45^hi^ cells.

### Statistical tests

Groups with normally distributed data points were compared using parametric unpaired t tests, while groups with non-Gaussian distributions were compared using non-parametric Mann–Whitney tests. Survival differences were analysed using the Log-Rank test. Standard error of percent inhibition was calculated from individual mice compared to the mean parasitaemia level of the control group.

## Results

### Cys enhances the anti-plasmodial activity of different artemisinin molecules against blood stage malaria in vivo (*P. chabaudi*)

**Fig. 1:**
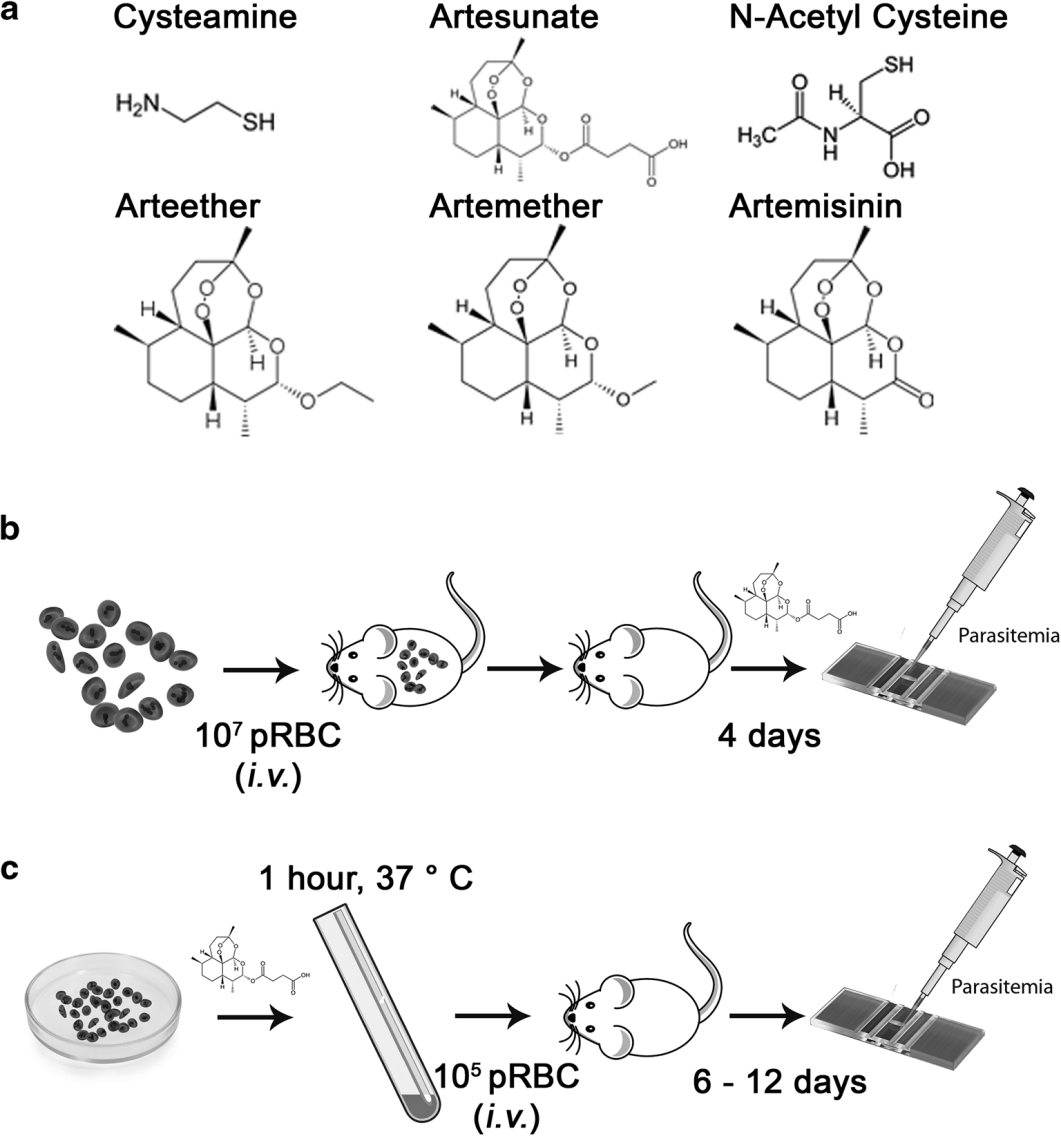
Experimental framework for testing anti-plasmodial activity of drug combinations. **a** Molecular structure of drugs used in the study. **b** Two types of infection protocols were used. For the in vivo*/*in vivo assay, mice were infected through the i.v. route with 10^7^ *Plasmodium* parasitized red blood cells (pRBCs). One hour later, drugs were administered through the i.p. route, parasitaemia was monitored on thin blood smears over time, and mortality was recorded. **c** For the ex vivo*/*in vivo assay, *Plasmodium* parasitized red blood cells were exposed for 1 h at 37 °C to different drug combinations. They were then washed free of drugs, counted and naïve mice were infected with 10^5^ treated pRBCs. Parasitaemia was monitored on thin blood smears over time, and mortality is recorded

The capacity of Cys to potentiate the anti-plasmodial activity of different ADs present in clinically-used ACT, including ART, ARTM, and β-ARTE (Fig. 1a) was investigated. ADs were given at sub-optimal concentrations (doses that have little effect on parasitaemia and survival on their own) to distinguish between the lack of an effect and possible additive or synergistic effects of Cys on the ADs, as previously described [26]. Synergy is defined as a total activity of the two compounds given together being greater than the sum of the independent activities of the two compounds given alone. The effect of Cys on ADs anti-plasmodial activity was determined using a standardized four-day test using blood parasitaemia as a quantitative measure of anti-plasmodial activity (Fig. 1b). In these experiments, mice were infected with *P. chabaudi* (10^7^ pRBC through i.v. route), and 1 h later, they received either PBS, Cys (140 mg/kg) and/or sub-optimal doses of ADs (between 0.05 or 0.5 mg/kg) given i.p. Parasitaemia levels were subsequently measured on day 4 and survival time was monitored.

**Fig. 2:**
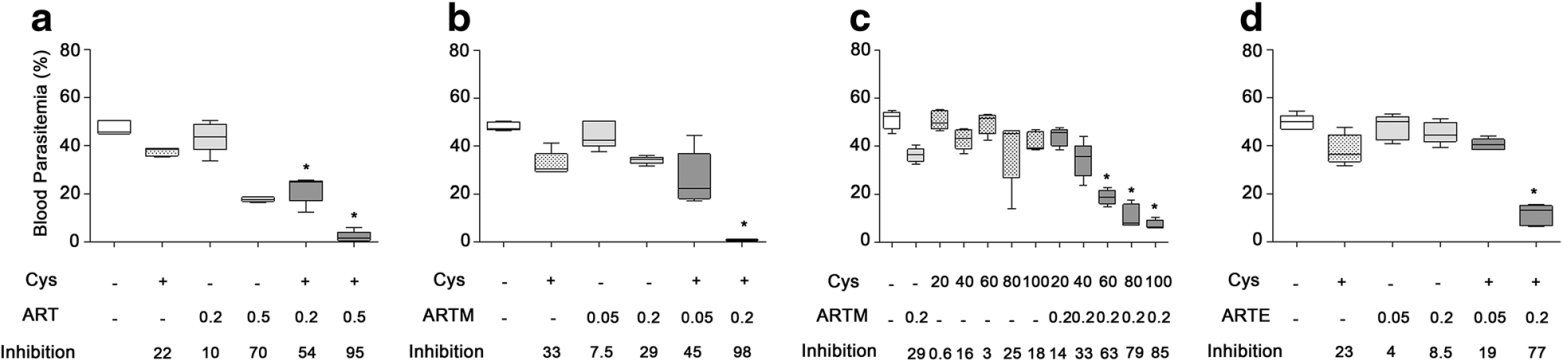
Potentiation of anti-plasmodial activity of artemisinin derivatives by Cys in *Plasmodium chabaudi* AS blood-stage infection (in vivo assays). Groups of A/J mice (minimum of 5 mice per group) were infected with *P. chabaudi* pRBCs, and were treated with different drug regimens at the indicated doses (in mg/kg). Cys dose was fixed at 140 mg/kg in *panels* **a**, **b**, and **d**, and at variable doses (20, 40, 60, 80, 100 mg/kg) in *panel* **c**. Artemisinin derivatives tested were ART (**a**), ARTM (**b**, **c**), and ARTE (**d**). The intensity of infection (blood parasitaemia) was measured at day 5 post-infection and is shown as* whisker plots* (interquartile ranges; mean, with minimum to maximum). The inhibitory activity of different drug treatments is shown as a fraction (% inhibition) of parasitaemia detected in control mice treated with PBS. Statistical significance was calculated by unpaired student t test, *p < 0.05. The data is representative of three independent experiments

For ART (Fig. 2a), sub-optimal dosing of 0.2 mg/kg (ART0.2) caused a modest ~10 % reduction of parasitaemia compared to untreated controls, while a higher dose (0.5 mg/kg; ART0.5) produced ~70 % inhibition; Addition of Cys to these two regimens resulted in further strong reduction of parasitaemia, with either a synergistic (ART0.2/Cys) or additive effect (ART0.5/Cys) (Fig. 2a). In addition, mice receiving both ART/Cys showed fewer symptoms of disease, and had a significant increase in survival time: from day six post-infection for control mice (PBS) and mice receiving either Cys alone or ART low dose (ART0.2), to day 7 for those receiving ART high dose (ART0.5) and the ART0.2/Cys combination, to day 10 for mice receiving ART0.5/Cys (Additional file 1A). For ARTM, the strongest effect on parasite replication was seen for the ARTM (0.2 mg/kg)/Cys combination (98 %), compared to Cys (33 %) or ARTM (29 %) alone (Fig. 2b). The ARTM (0.2 mg/kg)/Cys combination produced a synergistic effect and completely blocked parasite replication (<1 % parasitaemia at day 4), and completely protected against lethality with 100 % survival of mice in this group compared to mice receiving either PBS, Cys or ARTM alone (Additional file 1B). Dose–response studies (Fig. 2c) showed that the potentiating effect of Cys was dose-dependent, and that a minimum dosing of 60 mg/kg was required for clear potentiation of ARTM effect on blood parasitaemia. Maximum potentiation was seen at 80 and 100 mg/kg Cys (Fig. 2c), with animals in these two groups surviving past day 7 (Additional file 1C). Cys was also able to potentiate the anti-plasmodial activity of ARTE; while single dosing of Cys (140 mg/kg) or ARTE (0.05; 0.2 mg/kg) caused a maximum reduction of ~20 % in blood parasitaemia, the addition of Cys to high dose ARTE caused a nearly ~80 % reduction in parasitaemia (Fig. 2d), and increased survival to day 8 (Additional file 1D) compared to PBS-treated controls (100 % mortality by day 5). Similar to the effect of Cys on ARTM activity, potentiation of ARTE was dose-dependent, with optimal effects seen at a dose of 100 mg/kg Cys (Additional file 2).

Taken together, these results (representative of three independent experiments) indicate that Cys can potentiate the anti-plasmodial activity of chemically distinct artemisinin molecules in vivo, reducing blood stage parasite replication and increasing survival following acute infection.

### Cys enhances the anti-plasmodial activity of artemisinin against blood stage malaria ex vivo (*P. chabaudi*)

**Fig. 3:**
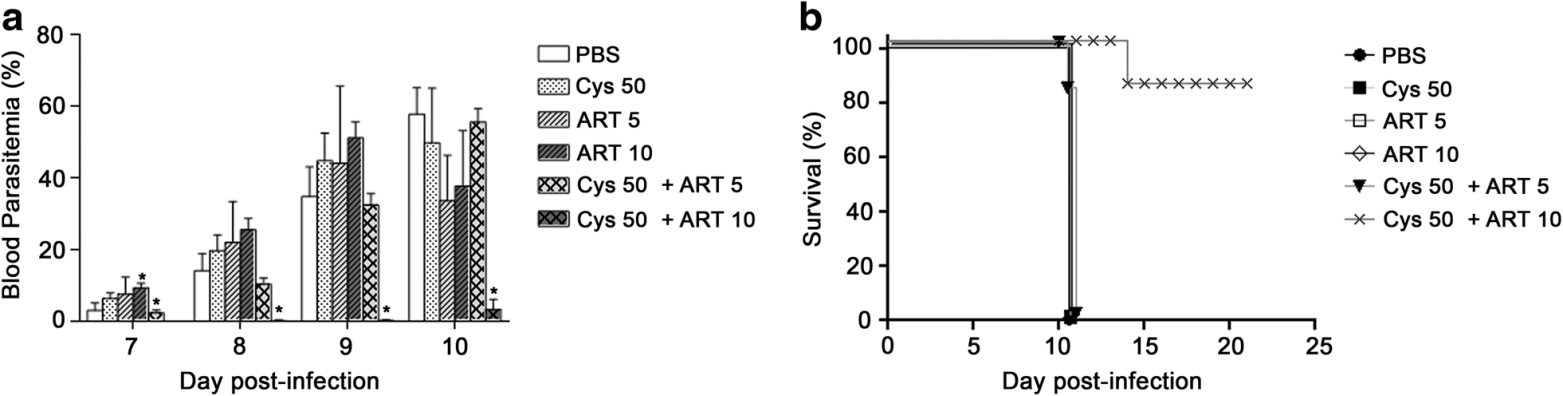
Potentiation of anti-plasmodial activity of ART by Cys in *Plasmodium chabaudi* blood-stage infection (ex vivo*/*in vivo assay). *P. chabaudi* parasitized red blood cells (pRBC) were exposed to different drug combinations in vitro for 1-h at 37 °C, washed free of drugs and then used to infect naïve mice. Groups of A/J mice (minimum of 5 mice per group) were infected with 10^5^ drug-treated *P. chabaudi* pRBCs, and infected mice were monitored for blood parasitaemia (**a**) at the indicated times post-infection (days 7–10). Drug concentrations are in micromolar (µM). Mortality was recorded (**b**). Data is representative of three independent experiments and it is expressed as mean ± SD for each group (*p < 0.05)

A series of experiments were conducted to determine whether the Cys potentiation of ADs anti-plasmodial activity noted against *P. chabaudi* in vivo is due to (a) increased drug induced anti-plasmodial activity, or (b) indirect static or cidal mechanisms involving the parasite replication niche (RBC) and/or host innate defenses. For this, *P. chabaudi* pRBCs were exposed to drugs in vitro, and the effect of this exposure on the ability of the treated preparations to induce infection in a naïve host in vivo was tested (Fig. 1c). *Plasmodium chabaudi* pRBCs were exposed for 1 h at 37 °C to either PBS, Cys (50 μM), ART (5, 10 μM) or both, which was subsequently washed off; The equivalent of 1 × 10^5^ pRBCs (calculated prior to drug exposure) was then injected into mice without any additional exposure to drugs in vivo, and parasite replication was followed over time (Fig. 3a). Results show that although a 1 h treatment with Cys alone or with ART alone (at both doses) had minimal effect on time of appearance and extent of blood stage replication, parasites treated with Cys (50 μM)/ART(10 μM) combination showed (a) considerable delay in onset of parasitaemia, (b) striking >90 % inhibition of parasite replication measured at day 9 and day 10, compared to PBS treated controls, and (c) significant survival (80 %) to infection compared to all other treatment groups which succumbed to infection by day 10 (Fig. 3b). These results indicate that Cys can strongly potentiate the anti-plasmodial activity of ART in a single 1 h exposure of pRBCs, significantly reducing parasite viability. The Cys enhancement of anti-parasitic activity is detected ex vivo, in the absence of the host immune system, strongly pointing at a direct enhancement of ART potency.

### Specificity of the Cys effect

**Fig. 4:**
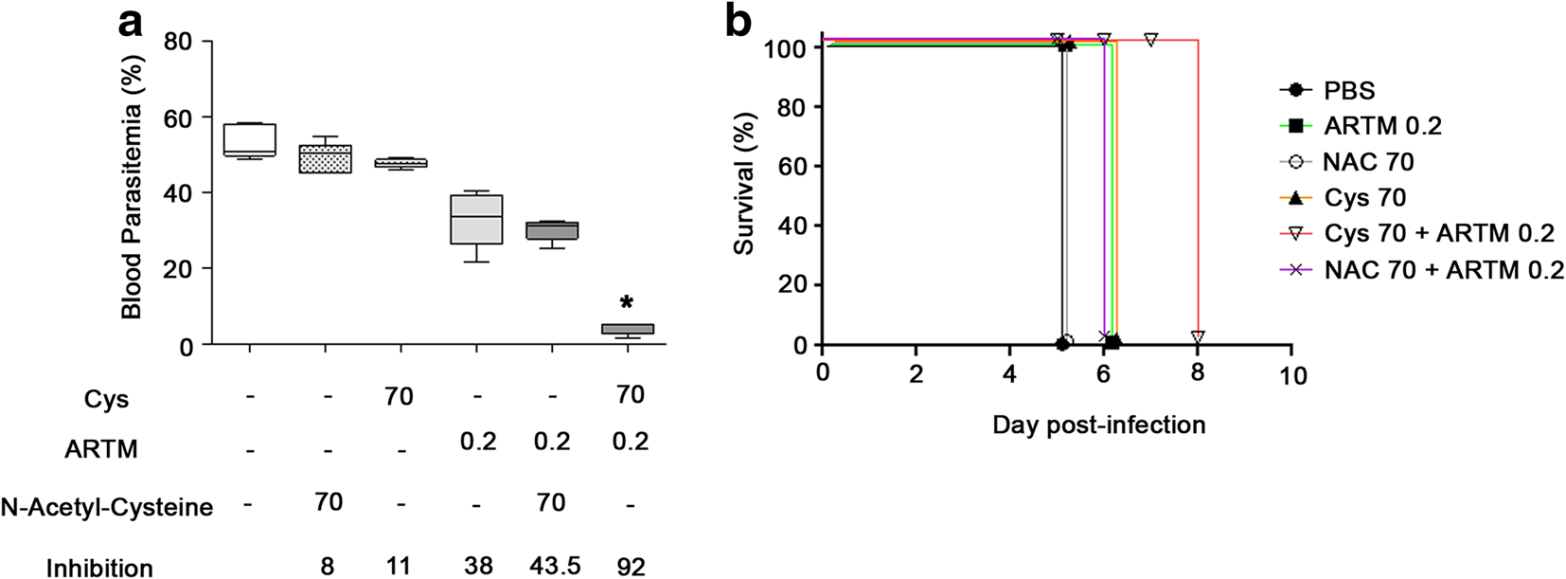
Specificity of Cys versus N-acetyl-Cysteine in potentiation of ARTM in *Plasmodium chabaudi* blood-stage infection. Mice (minimum of 5 mice per group) were infected by 10^7^ *P. chabaudi* pRBC through the i.v. route, and blood parasitaemia was monitored (**a**), and mortality was recorded (**b**), as described in legend of Fig. 2. The results show that at equivalent concentrations, Cys (70 mg/kg) can strongly potentiate the anti-plasmodial activity of ARTM (0.2 mg/kg), while NAC (70 mg/kg) has no effect. Statistical significance was calculated by unpaired student t test, *p < 0.05. Data are from one of two independent experiments that produced similar results

Cys is a naturally occurring amino-thiol that contains a thiol group which confers anti-oxidant properties (Fig. 1a). To determine whether the potentiating effect of Cys on artemisinin anti-plasmodial activity is specific, Cys was compared to another closely related aminothiol with similar anti-oxidant properties, N-acetyl-Cysteine (NAC; Fig. 1a). Mice were infected by 10^7^*P. chabaudi* pRBCs, and the infected mice were treated with either PBS, ARTM (0.2 mg/kg), Cys (MW 113) or NAC (MW 163) used at the same dose (70 mg/kg) alone or in combinations with ARTM (Fig. 4). The effect of drugs on blood stage replication of *P. chabaudi* was measured on day four post-infection (Fig. 4a). Results show that at the 70 mg/kg dose used, neither Cys alone nor NAC alone had a significant effect on *P. chabaudi* replication, and at the sub-optimal dosing of 0.2 mg/kg tested, ARTM had only a moderate effect on blood parasitaemia on day 4. As expected, the Cys/ARTM combination showed potentiation and caused a >90 % inhibition of parasite replication. By contrast, adding NAC to ARTM did not improve the anti-plasmodial effect of ARTM (~40 % inhibition). Cys/ARTM also improved survival to infection (up to 8 days), compared to mice from all other groups and that succumbed from infection between day 5 and 6 (Fig. 4b). Therefore, potentiation by Cys is specific and cannot be achieved with NAC.

### Cys enhances the anti-plasmodial activity of artemisinin against cerebral malaria (*P. berghei*)

**Fig. 5:**
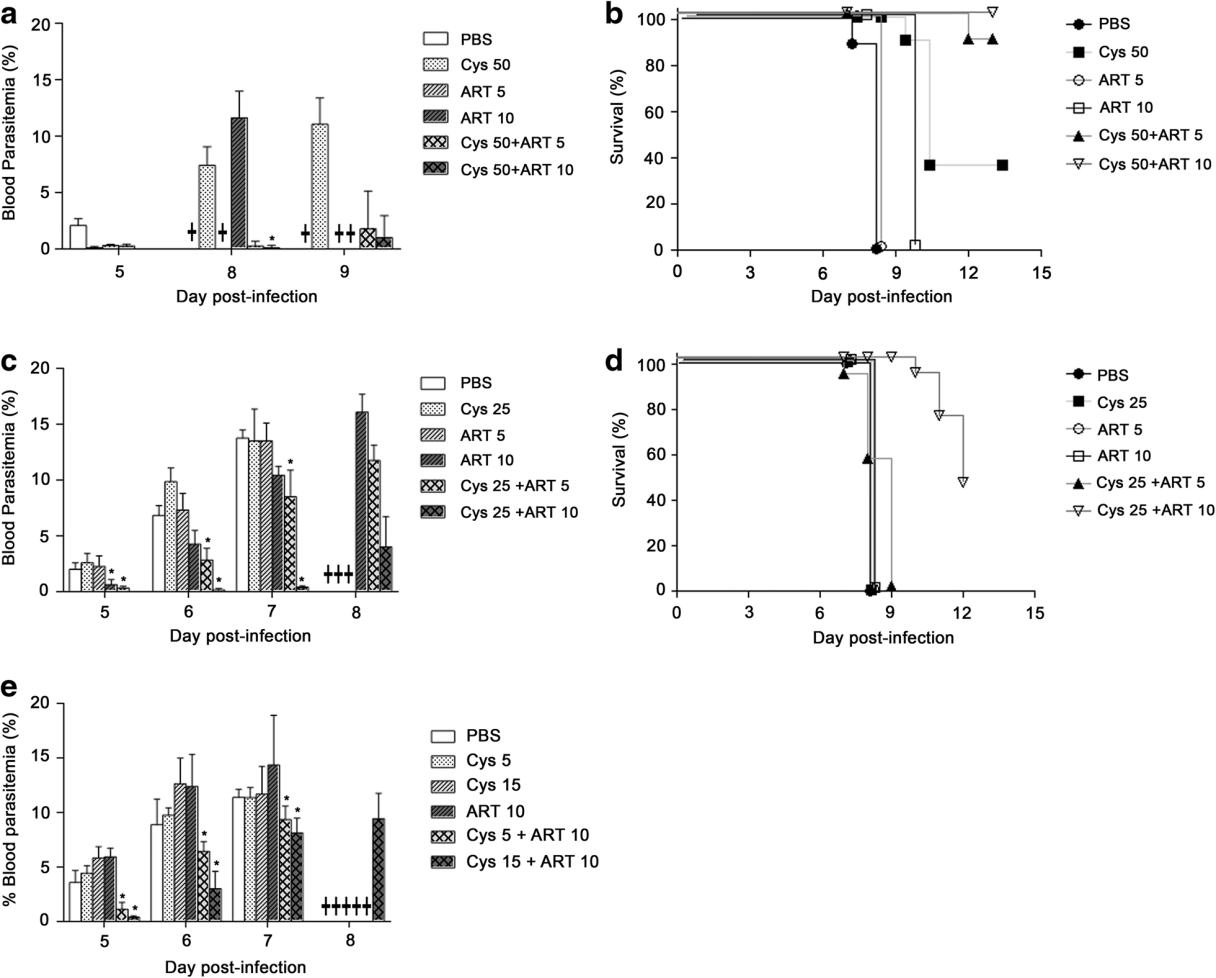
Potentiation of anti-plasmodial activity of ART by Cys in *Plasmodium berghei* cerebral malaria (ex vivo*/*in vivo assay). *P. berghei* pRBCs were exposed to different drug combinations (indicated in micromolar, µM) in vitro for 1 h at 37 °C (see Fig. 1). They were then washed free of drugs and used to infect naïve mice. Groups of C57BL/6J (B6) mice (minimum of 5 mice per group) were infected with 10^5^ drug-treated *P. berghei* ANKA-pRBCs, and monitored for blood parasitaemia (**a**, **c**, **e**) at the indicated times post-infection, and mortality was recorded (**b**, **d**). The potentiation of ART by decreasing concentrations of Cys, 50 micromolar (**a**, **b**), 25 micromolar (**c**, **d**), 15 and 5 micromolar (**e**) was tested. Mortality on *panels* **a**, **c**, **e** is indicated by a *cross*. Data is expressed as mean ± SD for each group (*p < 0.05). The data is representative of three independent experiments

Experimental cerebral malaria (ECM) can be induced in mice by infection with *P. berghei*. In this model, clinical symptoms occur at relatively low levels of blood parasitaemia (<20 %), and are not driven by haemolytic anaemia, but rather, by host pathological inflammatory reactions. Indeed, brain endothelial cells activated by pRBCs produce cytokines and chemotactic factors that recruit neutrophils and activated T lymphocytes. Release of cytotoxic molecules by inflammatory leukocytes leads to loss of integrity of the blood–brain barrier, micro-thrombosis, hypoxia of the brain parenchyma, leading to neurological symptoms including seizures and coma, and ultimately death [21, 22]. To determine whether Cys could be of therapeutic benefit against cerebral malaria, its capacity to potentiate the anti-plasmodial activity of ART against *P. berghei* was tested using the ex vivo exposure: in vivo infection model (Fig. 1c). Briefly, *P. berghei*-parasitized RBCs were exposed to different Cys/ADs combinations for one hour at 37 °C, and 10^5^ of the treated pRBC were injected into naïve mice. These were then followed for daily blood parasitaemia, for appearance of clinical symptoms, and survival was also recorded. Treatment of pRBCs in vitro with combinations of Cys (50 μM) plus ART (5, 10 μM) caused a significant delay in the onset of parasitaemia, and on the extent of parasite replication compared to pRBCs treated with either PBS, or with either Cys or ART alone (Fig. 5a). There was also a dramatic increase in the survival of mice infected with pRBCs treated with either Cys/ART combinations (80–100 % survival beyond day 13) (Fig. 5b). The enhanced anti-parasitic activity of the Cys/ART combination over either drug alone used to treat pRBCs in vitro was still evident when dosing as low as 25 μM Cys (Fig. 5c, d) or even 15 μM (Fig. 5e) were used. Together, these results (representative of 3 independent experiments) show that (a) Cys alone has significant activity against *P. berghei*; (b) Cys can strongly potentiate the anti-plasmodial activity of ART in the experimental cerebral malaria model; and (c) potentiation by Cys occurs at doses that are below those currently approved for life-long clinical treatment of cystinosis in humans.

**Fig. 6:**
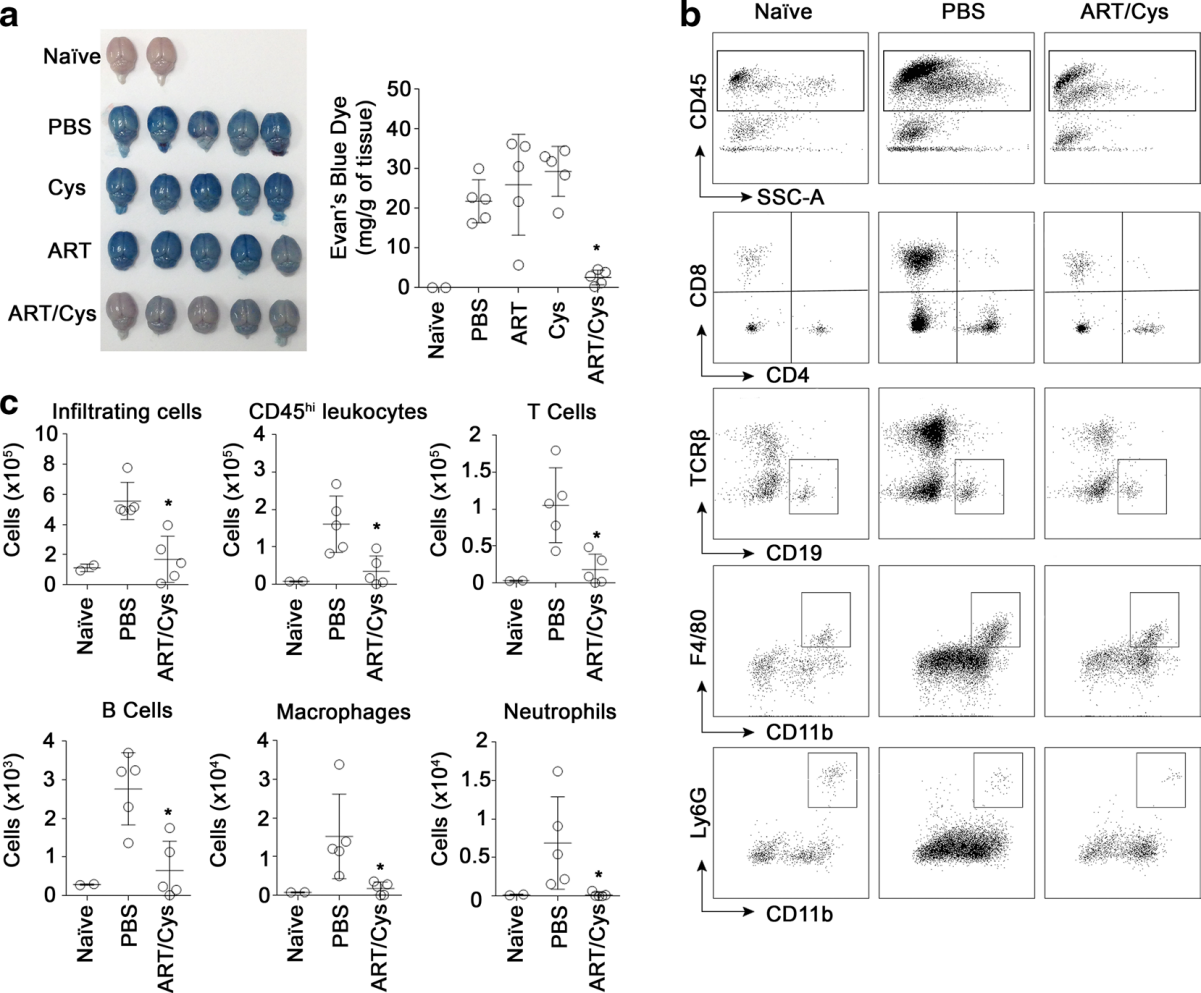
Reduced cerebral pathogenesis in mice infected with *Plasmodium berghei* treated with Cys/ART combination (ex vivo*/*in vivo assay). **a** Evans blue extravasation was used to assess integrity of the blood–brain barrier (BBB) of mice infected with *P. berghei* pRBCs treated ex vivo with either PBS, cysteamine (Cys) or artesunate (ART) alone, or in combination (ART/Cys) [see legend to Fig. 4]. Six days post-infection, mice were injected with Evans* blue dye*, the brains were harvested and photographed, and dye accumulation was quantified. Data is expressed as mean ± SD for each group (*p < 0.05). **b**, **c** Six days following *P. berghei* infection, infiltrating leukocytes were isolated by centrifugation on a 33.3 % Percoll gradient from brain homogenates and were analysed by FACS. **b** Representative FACS plots of cellular infiltration in the brain indicate reduced infiltration of CD45^hi^ leukocytes, CD45^+^CD4^+^ T cells, CD45^+^CD8^+^ T cells, TCRb^−^CD19^+^ B cells, F4/80^+^CD11b^+^ macrophages, and Ly6G^+^CD11b^+^ neutrophils in brains of mice that received ex vivo-exposed ART/Cys *P. berghei* parasites. **c** Data are expressed as the total number of viable cells in the brain, expressed as mean ± SD for each group (*p < 0.05)

The integrity of the blood brain barrier (BBB) of mice infected with *P. berghei* parasites (pRBCs) treated ex vivo with either PBS, Cys, ART or ART/Cys was also investigated, using an Evans blue extravasation assay [30, 31]. For this, mice at day six post-infection are injected with Evans blue, and the dye is allowed to circulate for 1 h prior to sacrifice. Brains of the mice were harvested, and the Evans blue dye was quantified (Fig. 6a). Mice infected with Cys/ART-*treatedP. berghei* displayed preserved BBB integrity with significant dye exclusion, and this was clearly distinct from mice infected with *P. berghei* pRBCs treated with either PBS, Cys, or ART alone which demonstrated compromised BBB function (Fig. 6a). In parallel experiments, *P. berghei*-infected mice were sacrificed at day 6 post-infection, exsanguinated, and perfused with PBS; isolated cells from the brain were analysed by fluorescence activated cell sorting (FACS) for the presence and number of infiltrating inflammatory cells (Fig. 6b, c). Mice infected with *P. berghei* parasites treated with Cys/ART combination showed reduced infiltration of total CD45^hi^ leukocytes, including macrophages (CD11b^+^F4/80^+^), neutrophils (CD11b^+^Ly6G^+^), CD19^+^ B lymphocytes, and CD4^+^ and CD8^+^ T lymphocytes (TCRβ^+^) (Fig. 6b, c). Taken together, these results indicate that improved clinical symptoms and overall survival in mice infected with *P. berghei* parasites treated with Cys/ART is associated with improved BBB function, reduced infiltration of pro-inflammatory myeloid and lymphoid cells, and absence of cerebral pathology.

## Discussion

In previous reports from our group, Cys was shown to have low but significant anti-plasmodial activity, and could also potentiate ART in an in vivo model of blood-stage malaria with *P. chabaudi* [27]. Building on these initial results, the current studies demonstrate that (a) the effect of Cys is broad, as it is found to potentiate the anti-plasmodial activity of structurally distinct artemisinin drugs in current clinical use in ACTs, (b) Cys increases the anti-plasmoidal activity of ART against both blood stage and cerebral forms of malaria, and (c) the Cys effect is direct, does not require host factors, and can be demonstrated on isolated *Plasmodium*-infected erythrocytes.

Cys can strongly potentiate the anti-plasmodial activity of the artemisinin derivatives ART, ARTM and ARTE. Artemisinin is itself a poor water-soluble and unstable sesquiterpene lactone that is not generally used clinically. ART, ARTM, ARTE, and dehydroartemisinin (DHA) are structural analogs of artemisinin that are in clinical use in current ACTs, including DHA-piperaquine (Artequick), ART-mefloquine, ART-amodiaquine, ART-sulfadoxine-pyrimethamine, ART-sulfamethoxypyrazine-pyrimethamine, and ARTM-lumefantrine [4, 5, 32]. In a standard four-day test in mice, Cys can strongly potentiate the anti-plasmodial activity of ART, ARTM, and ARTE against the blood stage parasite *P. chabaudi*, as determined by strong reduction of blood parasitaemia, and increased survival from acute infection. The observed effect of Cys on ART, ARTM, and ARTE suggests a potentiation mechanism that is independent of side chains substitutions of the main sesquiterpene lactone. Importantly, these results suggest that inclusion of Cys to current artemisinin-based anti-plasmodial drug formulations could improve efficacy of several ACTs. Furthermore, Cys potentiation of the anti-plasmodial activity of ART is observed for *Plasmodium* parasites that cause different pathologies, namely haemolytic anaemia associated with blood-stage replication of *P. chabaudi* AS, but also cerebral malaria associated with neuroinflammation caused by trapping of *P. berghei* parasitized erythrocytes at the blood brain barrier [23, 24, 26]. Hence, it is tempting to speculate that Cys may prove beneficial against different forms of malaria in humans.

What is the mechanism by which Cys potentiates the anti-plasmodial activity of artemisinins? This question remains unanswered. However, the present study shows that Cys potentiation may affect several aspects of the pathophysiology of *Plasmodium* infection. First and foremost, in vitro studies show that Cys can directly enhance the anti-plasmodial activity of artemisinins (in a 1 h assay), and without involvement of host-based mechanisms: Indeed, erythrocytes parasitized with different *Plasmodium* species (*P. chabaudi*, *P. berghei*) and exposed to the Cys/artemisinin combinations show significantly reduced capacity to induce a productive infection in naïve hosts (reduced blood parasitaemia and clinical symptoms, and increased survival). The observation that in this 1 h exposure assay, Cys can potentiate artemisinin activity, reducing infectivity and improving clinical endpoints (lethality) of *Plasmodium* parasite infections that involve different pathogenesis and causes of death (severe anaemia, encephalitis) support a direct increase in anti-parasitic property of the artemisinin/Cys combination. This proposal is supported by studies showing that Cys has modest but significant anti-plasmodial properties in vivo [24, 26].

In addition, Cys may have additional effects in vivo that may further enhance the anti-plasmodial properties of artemisinins. Indeed, in a parallel set of experiments, Cys pre-treatment was observed to affect pharmacokinetics of plasma ART, seemingly delaying the time of peak plasma concentration, from 5 min in controls (no Cys) to 10 min in Cys-treated mice (Additional file 3A, B), although there did not seem to be an effect on bioavailability (area under the curve; AUC). Cys also appeared to have an effect on the bio-transformation of ART to its biologically active species, dehydroartemisinin (DHA) [33, 34]. Results in Additional file 3C, D show that levels of plasma DHA were significantly higher at 10 and 20 min in the groups receiving Cys than in the group receiving ART alone, suggesting longer persistence of DHA in animals receiving Cys. These results suggest that in addition to the direct potentiation of ART anti-plasmodial activity (Figs. 2, 3, 4), Cys treatment in vivo may favorably modify the pharmacokinetics and metabolism of artemisinin to its active form DHA (enhanced transformation, and increase in plasma AUC), further increasing its anti-parasitic activity.

The mechanistic basis of the observed in vitro and in vivo effects remain to be fully elucidated but may additionally involve the anti-oxidant properties of the thiol group of Cys. Indeed, anti-oxidants have been proposed to improve malaria outcomes, but this has remained controversial. While some studies show that anti-oxidants may improve recovery from *Plasmodium*-induced oxidative stress [35, 36], other studies have not seen these effects, in fact arguing the opposite view as artemisinin plasmodial toxicity depends on in situ generation of free radicals which could be blunted by Cys [37, 38]. Because several of these studies used N-Acetyl Cysteine as an anti-oxidant, Cys and NAC were compared for potentiation of artemisinin in our experimental conditions. These studies showed that the Cys effect is specific, and that NAC has no activity when used in the same assay conditions (Fig. 4). This is supported by the findings that Cys anti-plasmodial activity is specific and not seen for other thiols such as dimercaptosuccinic acid (DMSA) [24].

Cys is a small aminothiol produced by the pantetheinase reaction which has very low toxicity, and various formulations of Cys have been approved for clinical use in the life-long management of nephropathic cystinosis, a rare pathology caused by mutations in the cysteine transporter [25]. For example, starting in early childhood, cysteamine bitartrate is given a following dose escalation schedule to reach a final dosing of ~2 g per day (divided into 4 daily doses) for a ~45–50 kg child. Continuous use of Cys at these doses over several years shows low toxicity in humans [39]. The pharmacokinetic profile of a single dose of 1.5 g of cysteamine bitartrate in humans (*C*_max_ = 80 μM; AUC = 2845.1 min μM) is similar to single subcutaneous (sc) injection of 50 mg/kg cysteamine hydrochloride in mice [26]. Here, Cys (cysteamine hydrochloride) can potentiate the anti-plasmodial potency of the artemisinin derivatives ART, ARTE and ARTM in vivo over a wide dose range, and at doses (40–60 mg/kg) that are well within the range of those used to treat human cystinosis. In addition, studies in vitro show that 1 h exposure to doses of Cys as low as 10–25 μM are sufficient to significantly potentiate anti-plasmodial activity of ART against both *P. chabaudi* and *P. berghei*. These Cys concentrations are well within the range of the *C*_max_ values (20–150 μM) reached by a single oral dose of cysteamine bitartrate used in humans [39–42]. The observations that (a) Cys is well tolerated and already in clinical use for chronic cystinosis, (b) it has anti-plasmodial activity of its own, (c) it can potentiate the effect of artemisinin derivatives currently in use, and at concentrations within the range of those currently in use for cystinosis treatment together strongly suggest that Cys has essential features of a new partner for artemisinin derivatives in current ACT.

Artemisinin included in ACT is currently the most important drug for the clinical treatment of malaria. Resistance to artemisinin in the malaria parasites would be a major threat to global health. Unfortunately, several reports have documented the recent emergence of artemisinin resistance, in particular at the Thai-Cambodia border area [12–16]. This has prompted the search for new therapeutic intervention, and new drug candidates with anti-plasmodial activity [43–48]. Results from the current study suggest that modification of current ACT by incorporation of Cys could represent a significant alternative for management of artemisinin-resistant *Plasmodium* malaria parasites.

## Conclusions

This report shows that Cys in combination with artemisinin can significantly improve the outcome of both blood-stage and cerebral malaria in mouse models of these two infections. In addition, the potentiating effect of Cys is broad and improves activity of several artemisinin derivatives used for clinical treatment of malaria in humans. In particular, the effect of Cys in the cerebral malaria model, where artemisinin is used as a single agent in humans, strongly suggests that its inclusion in management of these patients may significantly improve outcome. Cys potentiation might help overcome emerging artemisinin resistance in *Plasmodium* parasites and may widen its therapeutic activity. Taken together, these studies are a necessary pre-requisite to the clinical evaluation of Cys/ART combinations in humans.

## Additional files

10.1186/s12936-016-1317-3 Potentiation of anti-plasmodial activity of artemisinin derivatives by Cys in *Plasmodium chabaudi* blood-stage infection (in vivo assays): Effect on survival. Groups of A/J mice (minimum of 5 mice per group) were infected with *P. chabaudi* -parasitized red blood cells, treated with different combinations of Cys and artemisinin derivatives artesunate (ART; panel A), artemether (ARTM; panels B, C), and arteether (ARTE; panel D) at the indicated dosing (in mg/kg), and as described in the legend to Fig. 2.
10.1186/s12936-016-1317-3 Potentiation of anti-plasmodial activity of arteether by Cys in *Plasmodium chabaudi* blood-stage infection (in vivo assays): Cys dose–response. Groups of A/J mice (minimum of 5 mice per group) were infected with *P. chabaudi* -parasitized red blood cells, treated with different combinations of Cys and arteether (ARTE; 0.2 mg/kg) at the indicated dosing (in mg/kg), and parasitaemia was determined at day 4 post-infection, and as described in the legend to Fig. 2.
10.1186/s12936-016-1317-3 Effect of Cys on pharmacokinetics of ART in vivo. Mice were injected with Cys (140 mg/kg, i.p), and exactly 15 min later, ART (0.5 mg/kg, i.p) was injected. At the indicated times, mice were euthanized and bled by cardiac puncture and blood was collected in EDTA-containing tubes to isolate plasma. (**A, B**) The level of plasma artemisinin molecules (ART) was determined in two independent experiments. (**C, D**) Effect of Cys on biotransformation of ART to dihydroartemisinin in vivo is shown. The level of plasma dihydroartemisinin (DHA) was determined in two independent experiments. (**A-D**) Data is expressed as mean ± SD for each group (* *p* < 0.05).
