# Supplementary figures and images for: Cysteamine broadly improves the anti-plasmodial activity of artemisinins against murine blood stage and cerebral malaria
Source: Malar J. 2016 May 6;15:260. doi: 10.1186/s12936-016-1317-3 (PMC4858922; doi:10.1186/s12936-016-1317-3)

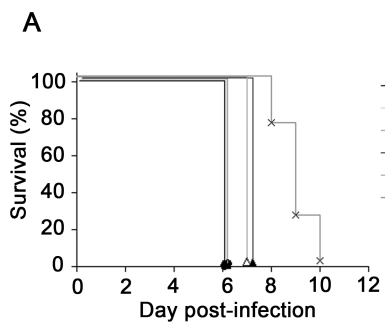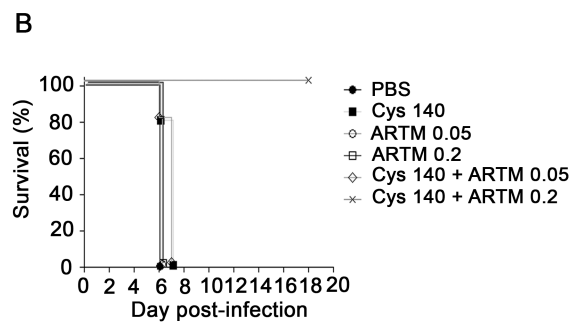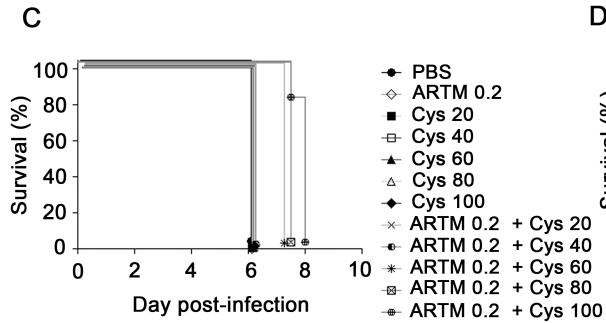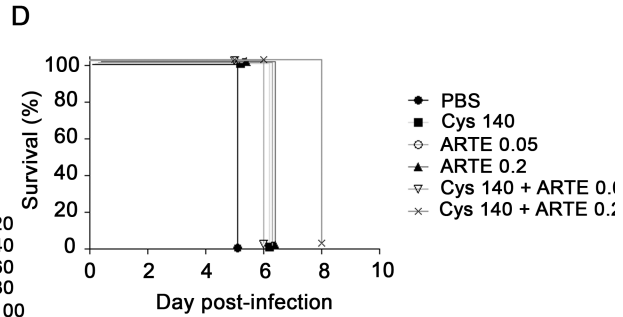

Supplement: Supplementary file 1 — 10.1186/s12936-016-1317-3 Potentiation of anti-plasmodial activity of artemisinin derivatives by Cys in Plasmodium chabaudi blood-stage infection (in vivo assays): Effect on survival. Groups of A/J mice (minimum of 5 mice per group) were infected with P. chabaudi -parasitized red blood cells, treated with different combinations of Cys and artemisinin derivatives artesunate (ART; panel A), artemether (ARTM; panels B, C), and arteether (ARTE; panel D) at the indicated dosing (in mg/kg), and as described in the legend to Fig. 2. [file 12936_2016_1317_MOESM1_ESM.pdf]

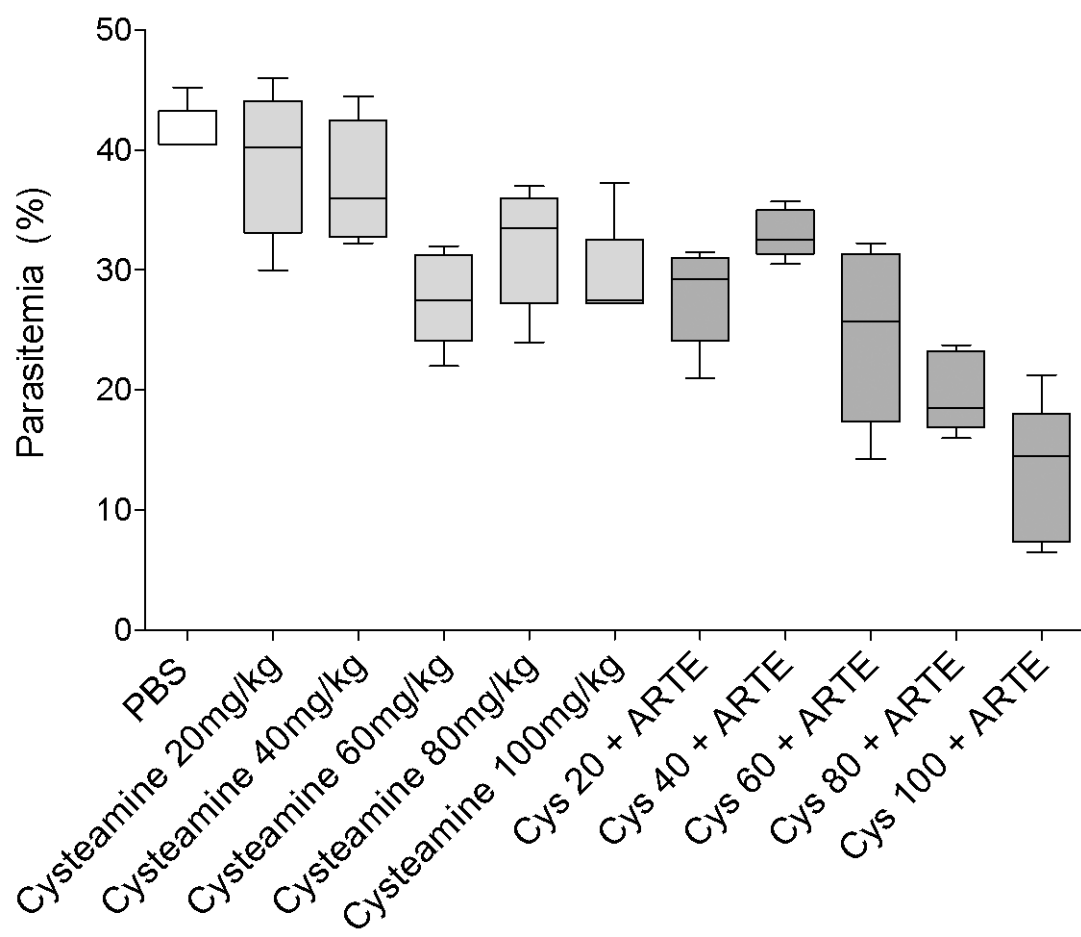

Supplement: Supplementary file 2 — 10.1186/s12936-016-1317-3 Potentiation of anti-plasmodial activity of arteether by Cys in Plasmodium chabaudi blood-stage infection (in vivo assays): Cys dose–response. Groups of A/J mice (minimum of 5 mice per group) were infected with P. chabaudi -parasitized red blood cells, treated with different combinations of Cys and arteether (ARTE; 0.2 mg/kg) at the indicated dosing (in mg/kg), and parasitaemia was determined at day 4 post-infection, and as described in the legend to Fig. 2. [file 12936_2016_1317_MOESM2_ESM.pdf]

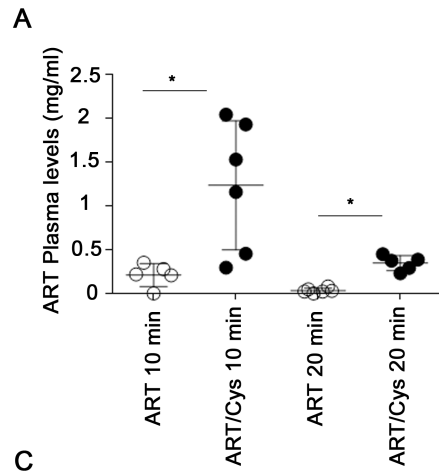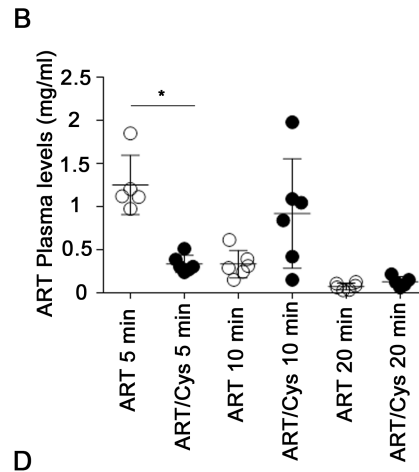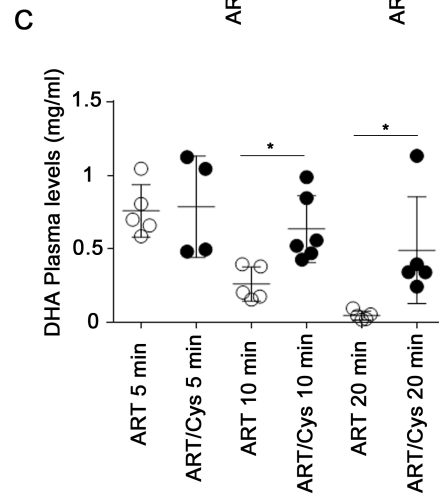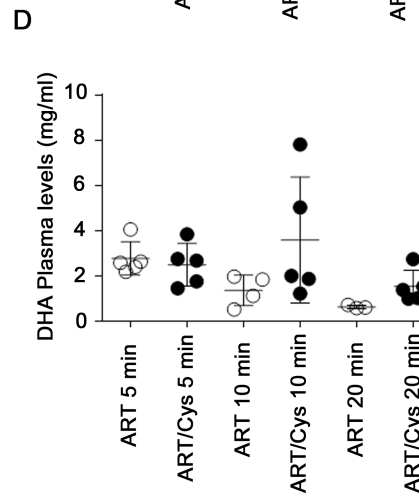

Supplement: Supplementary file 3 — 10.1186/s12936-016-1317-3 Effect of Cys on pharmacokinetics of ART in vivo. Mice were injected with Cys (140 mg/kg, i.p), and exactly 15 min later, ART (0.5 mg/kg, i.p) was injected. At the indicated times, mice were euthanized and bled by cardiac puncture and blood was collected in EDTA-containing tubes to isolate plasma. (A, B) The level of plasma artemisinin molecules (ART) was determined in two independent experiments. (C, D) Effect of Cys on biotransformation of ART to dihydroartemisinin in vivo is shown. The level of plasma dihydroartemisinin (DHA) was determined in two independent experiments. (A-D) Data is expressed as mean ± SD for each group (* p < 0.05). [file 12936_2016_1317_MOESM3_ESM.pdf]
